# Supplementary material for: Data-driven unsupervised clustering of online learner behaviour
Source: NPJ Sci Learn. 2019 Sep 3;4:14. doi: 10.1038/s41539-019-0054-0 (PMC6722089; doi:10.1038/s41539-019-0054-0)
Supplement: Supplementary file 1 — Supplementary Information [file 41539_2019_54_MOESM1_ESM.pdf]

Data-driven unsupervised clustering of online learner behaviour
Supplementary Information

Robert L. Peach,[1,3] Sophia N. Yaliraki,[2] David Lefevre[3] and Mauricio Barahona[1*]

[1]Department of Mathematics, Imperial College London, London, SW7 2AZ
[2]Department of Chemistry, Imperial College London, London, SW7 2AZ
[3]Imperial College Business School, Imperial College London, London, SW7 2AZ

1 Further details of multiscale clustering of the first Imperial Business School cohort

For completeness, we present here a more detailed representation of the multiscale clustering results of the first cohort of Imperial Business School learners studied in Fig. 3 of the main text. Supplementary Figure 1 shows the same quasi-hierarchical clustering of Fig. 3, but including the 8-way and 3-way partitions. Note how the green and black clusters in the 8-way partition agglomerate into the single green cluster in the 6-cluster partition, and one of the single learners (45) integrates into the cyan cluster. Beyond these two changes, there is little difference between the 8-way and 6-way partitions. As in the main text, the low and high performance learners are indicated by red and blue circles, respectively. In the 10-cluster partition, the purple cluster has a strong over-representation of low performers: 6 out of 7 low performance learners are found in the purple cluster, which contains 17 out of 81 learners in the full cohort. Hence this cluster is enriched 4-fold in low performers, as compared to expectation.

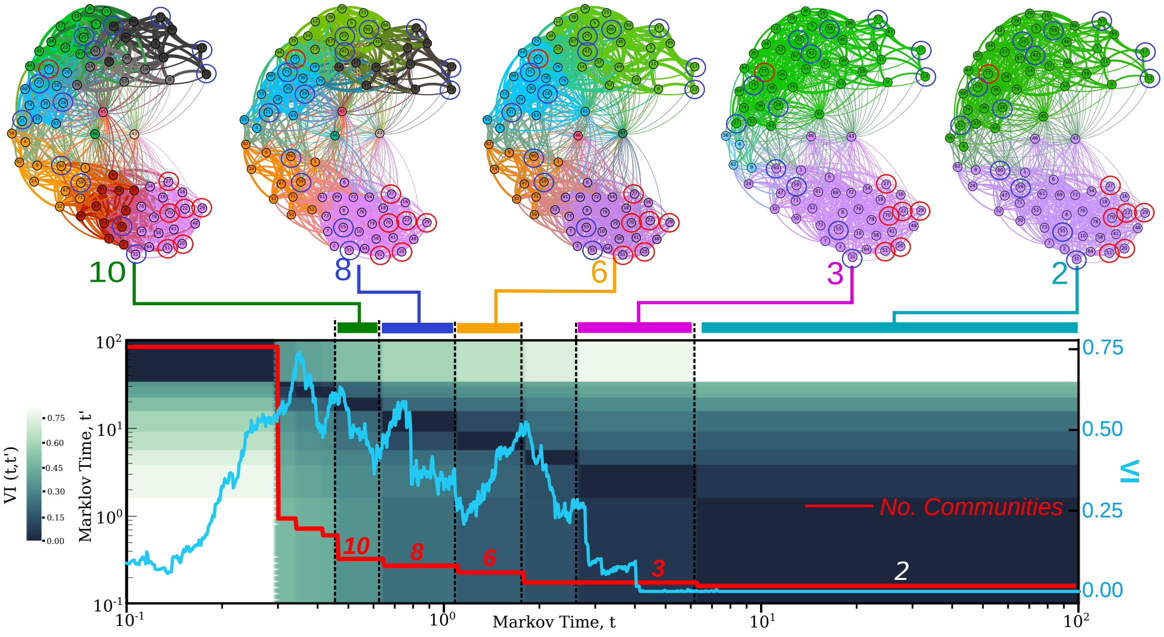

Supplementary Figure 1: Markov Stability multiscale clustering structure of the first cohort of Imperial Business students (same as in Figure 3 of the main text but with further detail of the 8-way and 3-way partitions). Red circles indicate low performers ($< 60\%$) and blue circles indicate high performers ($> 70\%$).

2 Application of the methodology to two additional examples

To test the applicability of the method further, we have applied the methodology to two additional datasets with different characteristics.

2.1 Second cohort of Imperial College Business School learners

We have collected further data from a second cohort of learners undertaking a similar online management course at Imperial College Business School. The subjects in this research were 46 post-experience learners pursuing a post-graduate part-time management degree. Detailed learner demographic information was anonymised. The data is of the same type as the one collected and analysed for the first cohort from the same source, i.e., the data corresponds to completed tasks for six online courses which together comprised the first academic year of the 2-year degree programme. Although the subjects met face-to-face at the start of each academic year, the six courses were studied completely online. Subjects proceeded in a lock-step manner through the academic year which was split into three 10-week terms each containing two of the six courses. The anticipated study load was 5 to 7 hours per week for each course, so 10 to 12 hours in total. The courses were assessed via a combination of coursework and exam. However, participation in these separate assessed activities was not included in the dataset analysed here, only their final 2-year grade was used as an indication of their performance.

The results of our analysis are shown in Supplementary Figure 2. We identify robust partitions into 9, 6 and 2 clusters based on the optimality and robust criteria discussed in Methods. The partitions identify major clusters and outlier clusters (single nodes in own cluster); for instance, the 6-way partition contains 3 major clusters and 3 outliers. The multi-scale clusterings are quasi-hierarchical: the clusters in the 9-way partition generally aggregate to form the coarser clusters in the 6-way partition and finally into the 2-cluster partition (Supplementary Figure 2A)

The temporal characteristics of the clusters of the 6-way partition are presented in Supplementary Figure 2B. The purple cluster corresponds to learners exhibiting massed learning, whereas learners in the cyan cluster exhibit less massed learning but still low task engagement. The green cluster has learners operating under distributed learning and high task engagement. The three outliers exhibit sporadic behaviours with localised patterns in time for particular parts of the course.

The other robust clusterings of different coarseness in the multiscale analysis provide additional information. For instance, the black cluster in the finer 9-way partition corresponds to 'Early birds', i.e., learners that complete tasks much earlier than those in the green cluster and with similar task completion statistics. For the coarser scale, the 2-cluster partition generally divides learners into a group that exhibits massed learning and low engagement (purple cluster) versus another group of learners with high engagement and distributed on-time or early task completion (green cluster).

We have also considered the performance of these learners *a posteriori*, as indicated with red circles (low performers, $< 60\%$) and blue circles (high performers, $> 70\%$) in Supplementary Figure 2. Again, we observe that the low performers tend to concentrate in the massed learning cluster (purple) or sporadic behaviours (e.g., in the 2-cluster partition, 7/8 low performers are in the purple cluster of massed learning/low task completion). As we found in the analysis of the first cohort, we also find that high performers are distributed across clusters with different temporal behaviours, although with a higher prevalence in the distributed learning cluster.

As we did for the first cohort, we have also trained two classifiers (a Support Vector Machine with Radial Basis Function kernel and a Decision tree) to use two statistical features of the time series to classify the learners into low/mid/high performance. Supplementary Figure 3 shows that the SVM and Decision Tree classify correctly 2/8 and 4/8, respectively, of the low performing learners.

The findings in this second cohort of Imperial Business School learners agree agree with our analysis of the

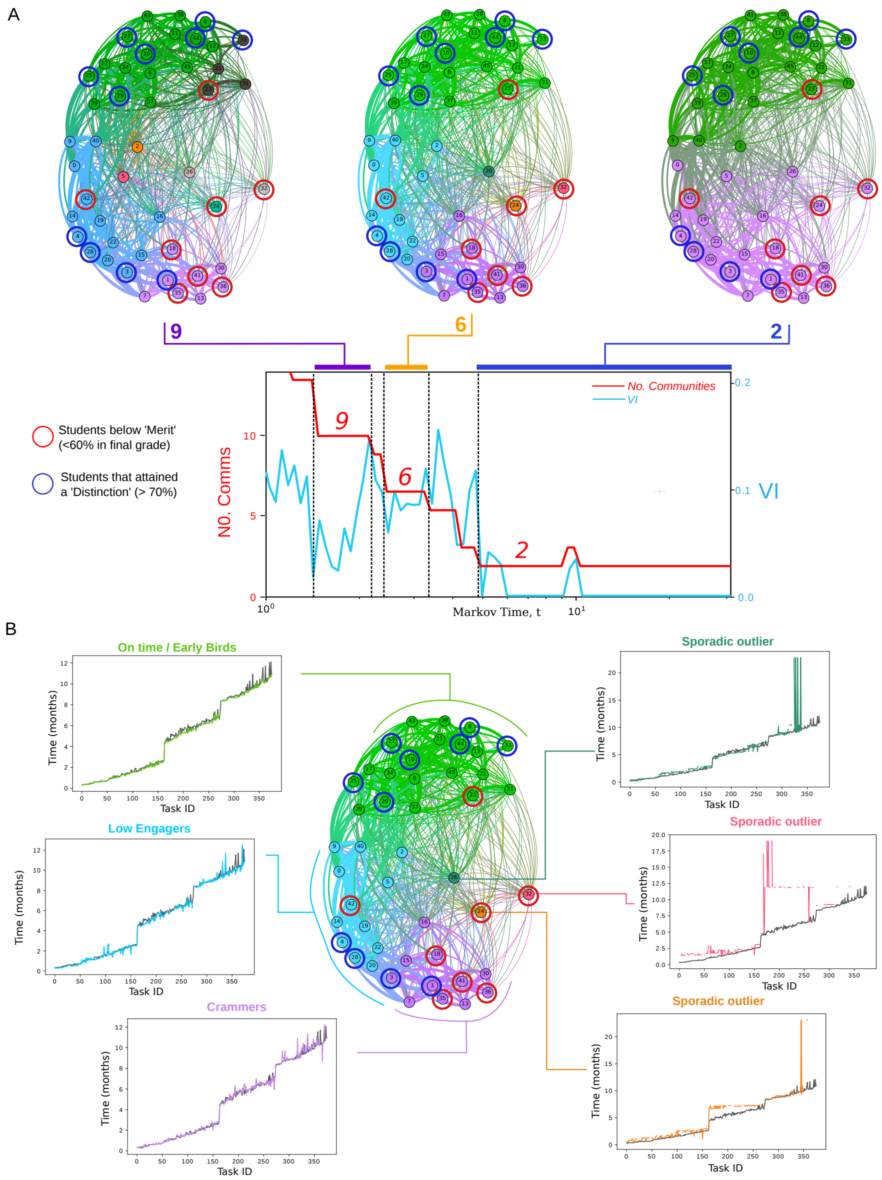

Supplementary Figure 2: Application of our methodology to a second cohort of learners ($N = 46$) undertaking a similar online management course at Imperial Business School with a different set of tasks (No. tasks = 376) over a 1-year period. *A*) The multiscale clustering reveals optimal and robust 9, 6 and 2-way partitions. The 9-way partition is dominated by 4 major clusters and 5 outliers; the 6-way partition is dominated by 3 major clusters and 3 outliers and show a quasi-hierarchical structure. *B*) Temporal behaviours associated with the 6-way partition analysed in more detail and the average GPR time-series of each cluster was plotted. As compared to the clusters obtained in the first cohort (Fig. 3 in the main text), the green cluster exhibits similar behaviour to the 'Early birds' and 'On time' groups (with distributed learning and low task skipping); the cyan cluster is similar to the 'Low engager' group (with considerable task-skipping and some massed learning); and the purple cluster is similar to the 'Crammer' group (with students displaying repeated massed learning and task skipping). We find that 6/8 of the low performance students (red circles) are found in the purple cluster or displayed highly sporadic engagement behaviour and only 1/8 is found in the green cluster of distributed learning behaviours. The high performers, on the other hand, are diverse in their patterns of online engagement, as also observed in the first cohort (Fig. 3 in the main text).

first cohor [illegible] ain text.

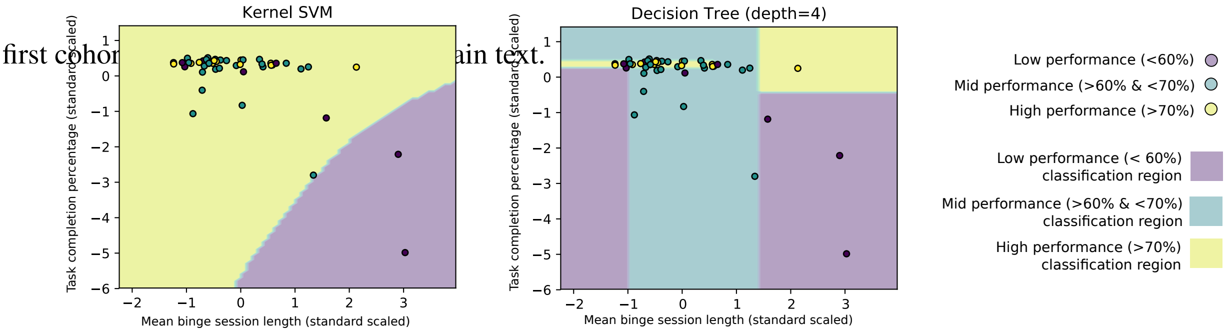

Supplementary Figure 3: Classification of learners with a trained SVM (RBF kernel) and a Decision tree (depth 4) using two statistical features of the time series (mean binge session length and task completion). Only 2/8 low performance learners are accurately classified using the SVM and 4/8 using the decision tree. Both statistical features are normalised to a z-score (subtracting mean and normalising to unit variance).

2.2 A set of learners from the Open University dataset (OULAD)

We have also applied the methodology to a set of learners of similar size ($N = 100$) extracted at random from the OULAD dataset of students undertaking the Open University courses (Kuzilek et al., 2017). There are significant differences between the OULAD dataset and the Imperial Business School datasets used in the main text (Figure 3) and in Supplementary Figure 2. Most notably, the time stamps in the OULAD dataset correspond to 'click data' on pages and do not necessarily relate to task completion, but rather reflect 'browsing of material'. It is therefore interesting to apply our method to time-stamped data with such qualitatively different characteristics.

Supplementary Figure 4 shows the results of our multiscale clustering analysis for the learners from the OULAD dataset. The figure also serves to exemplify the robustness of the method to the construction of the graph similarity using the Relaxed Minimum Spanning Tree (RMST) algorithm. As discussed in the main text, the similarity matrix is sparsified using the RMST and the level of sparsification is controlled with the parameter γ. Supplementary Figure 4 shows the resulting similarity graphs obtained from the same dataset as the sparsification is increased—as γ is reduced from 0.05 to 0.02, the number of edges drops from 3558 to 892 (out of a maximum 4950 possible edges in this dataset). The multiscale clustering associated with each graph shows that there is a robust optimised 3-way partition across the different levels of sparsification.

The 3 cluster partition contains two major clusters (purple and orange, corresponding to massed and distributed learning, respectively) and one minor cluster (green) that tends to include outlier learners. Note that 6/7 of the low performing learners (red circles) are consistently contained in the purple cluster of massed learning and the remaining one (1/7) falls in the minor cluster of sporadic behaviours (green). None of the low performing learners are found in the distributed learning cluster (orange). Furthermore, we found that the learners in the massed learning (purple) cluster have a significantly lower grade (measured by paired t-test with 5%) relative to the distributed learning (orange) cluster. This significant difference of grades between massed learning and distributed learning clusters is observed in all levels of network sparsification.

These results highlight the applicability of the approach and consistency of outcomes even when applied to time-stamped activity datasets of diverse origin and interpretation.

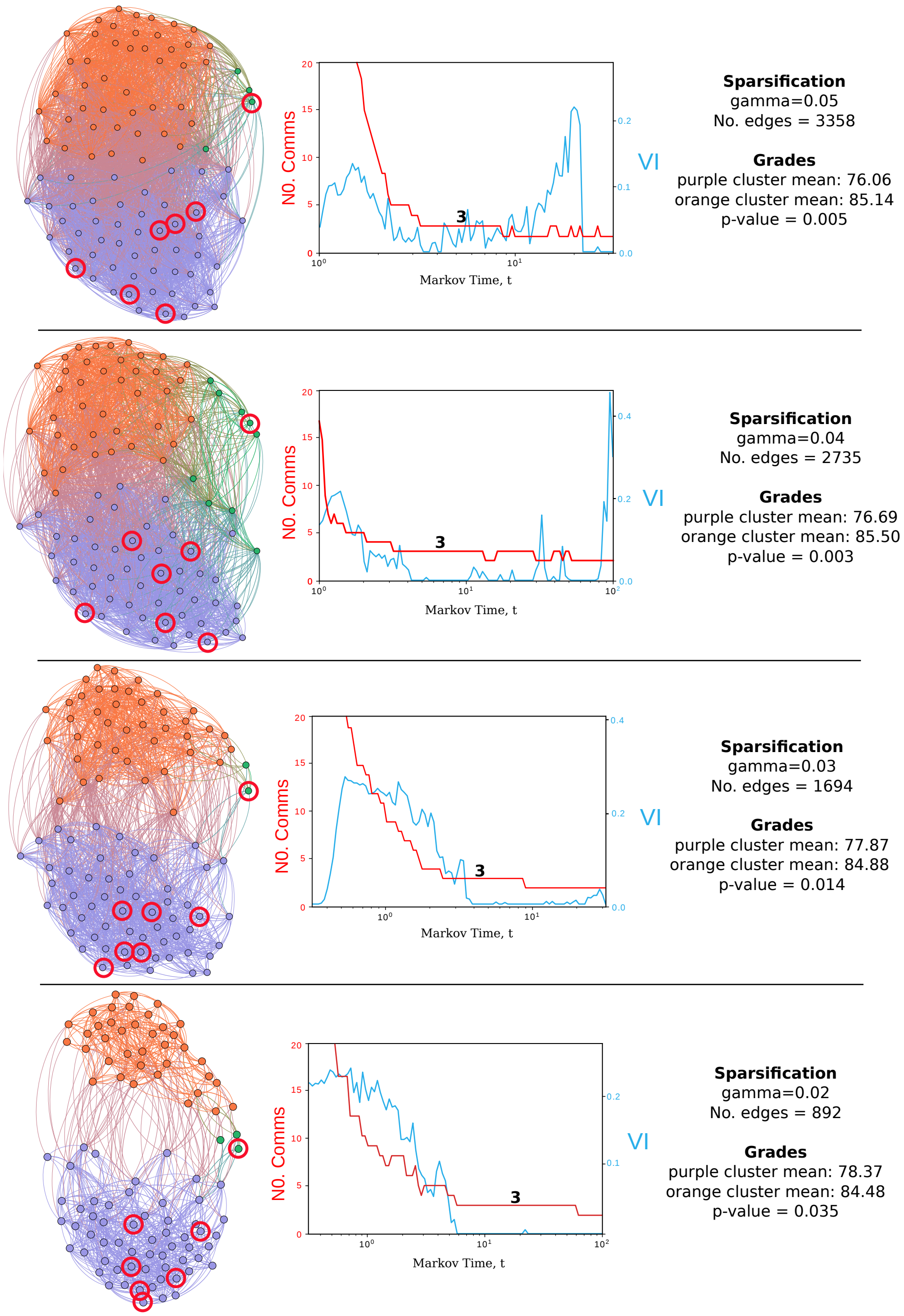

Supplementary Figure 4: Analysis of time-stamped data from a cohort of students undertaking an anonymised course in the OULAD dataset over 250 days (Kuzilek et al., 2017). The time series of this data set differ from those of the two cohorts of Imperial Business School students in that the time stamps correspond to ‘page clicks’ and therefore do not necessarily constitute task completions. Various levels of sparsification achieved with the RMST algorithm (with varying parameter γ) are used to show the robustness of the multiscale community detection methodology. The Markov Stability analysis finds a robust 3-cluster partition consistently across all level of sparsifications of the dataset. Within the 3-way partition, there are two major clusters (purple and orange) and one minor cluster (green) that tends to include outlier behaviours. The purple cluster corresponds to massed learning and contains 6/7 of the low performers and has a significantly lower grade (paired t-test at 5%) relative to the orange cluster of distributed learning behaviours. This difference exists at all levels of network sparsification.

References

Jakub Kuzilek, Martin Hlosta, and Zdenek Zdrahal. Data Descriptor: Open University Learning Analytics dataset. *Scientific Data*, 4:1–8, 2017. ISSN 20524463. doi: 10.1038/sdata.2017.171.
